# The Role of Title 1 Secondary School Athletic Trainers in the Primary and Patient-Centered Care of Low Socioeconomic Adolescents

**DOI:** 10.3390/ijerph20075411

**Published:** 2023-04-05

**Authors:** Nicolette A. Harris, Michelle L. Odai

**Affiliations:** 1 Department of Athletic Training, A.T. Still University, Mesa, AZ 85206, USA; 2 Department of Athletic Training, Florida International University, Miami, FL 33199, USA

**Keywords:** social determinants of health, access to care, primary care, integration of care, advocacy, patient-centered care, athletic trainer

## Abstract

Athletic trainers (ATs) provide regular encounters with a healthcare provider for many Title 1 student–athletes with healthcare access and quality barriers. Thus, they are uniquely positioned to serve as a student–athletes’ first point of contact for general medical concerns. This study aimed to describe ATs’ experiences providing primary care for Title 1 student–athletes. This qualitative design employing an interpretative phenomenological analysis (IPA) approach used in-depth, virtual focus groups to examine the experiences of ATs practicing at Title 1 secondary schools. The findings reveal that ATs were called upon to evaluate, treat, and, when necessary, refer student–athletes with general medical conditions. However, Title 1 ATs encountered numerous complex social determinants of health (SDoH) preventing efficient and effective referral to specialty healthcare providers. Thus, ATs ultimately felt their most important roles in the primary care of low socioeconomic adolescents were as caregivers who mitigated avoidable healthcare barriers in addition to coordinators of integrated care that assisted student–athletes and their families with navigating the healthcare system. Title I ATs need to be aware of the SDoH affecting their student–athletes and the ability of those SDoH to affect health outcomes as well as overall student–athlete health and well-being.

## 1. Introduction

Social determinants of health (SDoH) or the conditions wherein people are born, grow, live, learn, and work can significantly impact health outcomes. Individuals residing in low socioeconomic communities face barriers to overcoming these SDoH, particularly lack of health care access and quality [1]. Adolescents from lower socioeconomic backgrounds have been shown to have inferior health outcomes [2]. As many as one in eight adolescents lack a usual source for routine preventive care and as many as one in eleven adolescents report having no usual source of care when sick or injured [3]. This is due in part to health inequities suffered by these individuals, such as lack of insurance, underinsurance, greater reliance on public insurance, and lesser access to healthcare providers [4]. Heads of households report spending significant time searching for public health insurance [2], after which, when secured, policy holders report spending significant time searching for clinicians and facilities that accept their plans [2]. Further surmounting these obstacles, there is currently a shortage of primary care providers (PCPs) that is expected to only grow through to the year 2030 [5]. To address the challenges encountered by these medically underserved populations, creative use of healthcare resources will be required [5].

Although athletic trainers (ATs) are commonly perceived to treat only sport-related injuries and conditions, they have continued to expand their practice to be well-suited for addressing this healthcare challenge [5,6]. Several of the athletic training (AT) practice domains overlap with the family practice sub-competencies of the Accreditation Council for Graduate Medical Education (ACGME) [7]. For example, the first domain of AT entails risk reduction, wellness, and health literacy, which aligns aptly with the ACGME’s third patient care competency of partnering with the patient, family, and community to improve health through disease prevention and health promotion [5,7]. Likewise, the fourth athletic training domain of therapeutic intervention describes the AT’s ability to rehabilitate and recondition injuries as well as general medical conditions with the goal of achieving optimal activity levels [5]. This domain uses similar language to the fifth ACGME patient care competency by denoting both the family practitioners’ ability to perform specialty procedures to meet the healthcare needs of patients, families, and communities as well as their knowledge about the procedures performed by other healthcare professionals to guide their patients’ care [5,7]. Given this similarity, ATs should be viewed as a potential strategy for helping to alleviate the growing primary care shortage for adolescents in lower socioeconomic communities [5,6].

While still considered a luxury in some regions of the country, AT services are generally free of charge if provided to student–athletes at secondary schools. Therefore, for adolescents from lower SES backgrounds who have limited–to-no access to quality health care, interactions with ATs through interscholastic activities may be one of the only regular encounters they have with a healthcare provider [4,8,9]. This positions ATs ripely to serve as the first point of contact regarding general, non-orthopedic medical concerns and a vital bridge to the healthcare system for this vulnerable patient population [4]. Secondary school ATs can use patient-centered strategies to guide student–athletes to school-based or free community health centers and serve as advocates for these services in the event that they do not exist [9]. Therefore, the objective of this study was to describe the experiences of ATs providing primary care for adolescent student–athletes attending Title 1 secondary schools.

## 2. Materials and Methods

### 2.1. Research Design

To gain an understanding of the participants’ experiences providing primary care in Title 1 schools, we used a qualitative design consisting of in-depth, virtual focus groups which conceptualized the use of an interpretative phenomenological analysis (IPA) research approach. The IPA approach was constructed from Guba’s Critical Theory Paradigm (1990) in addition to Burrell and Morgan’s (1979) for the purpose of determining the impact of a problem or issue on the ‘lived experience’ of the research participants [10]. This study was approved for non-exempt human subject research by the Institutional Review Board (IRB) at Florida International University.

### 2.2. Instrumentation

A semi-structured interview guide was developed and used to assist investigators in exploring the experiences of ATs during focus groups. This protocol, comprising 5 open-ended questions and additional follow-up questions, was used to gain more information regarding the context of the athletic trainers’ experiences. Content and design experts were used to review the interview protocol for content validity. The interview guide can be found in Table 1.

### 2.3. Participants and Procedures

The use of an IPA design warranted a homogenous sample of approximately 12 participants with similar lived experiences [10,11]. Therefore, initial recruitment began in March 2021 through invitation letters that were sent to a convenience sample of secondary school ATs. When the targeted number of participants was not obtained, a snowball sampling method utilized qualified participants to recruit additional study participants meeting the inclusion criteria through July 2021 until data saturation was reached (*n* = 11). Participants were included if they were ATs practicing at Title 1 secondary schools. Title 1-A grants provide supplementary education and related services to schools, pre-kindergarten through to grade 12, with relatively high concentrations of students from low-income households [12].

Invitations to participate which detailed the study’s purpose, design, total time commitment, and incentives for participation were sent to a convenience sample of participants via GroupMe (New York, NY, USA) and Twitter (San Francisco, CA, USA). The principal investigator (N.A.H.) collected names, the secondary schools of practice, and school-issued email addresses from ATs expressing interest in study participation. The inclusion criteria were verified by the principal investigator (N.A.H.) through communication via the ATs’ school-issued email addresses. Current Board of Certification, Inc. (BOC) certification was also verified using the BOC website. Likewise, Title 1 school status was verified by the principal investigator (N.A.H.) by using the National Center for Education Statistics website at nces.ed.gov (accessed on 12 July 2021). Eligible participants were emailed via their school-issued email addresses with details regarding participation. Recruitment emails also provided a link to complete a survey via Qualtrics (Provo, UT, USA) that gathered participants’ demographic information. Electronic informed consent was provided at the beginning of the survey. A Doodle (Zürich, Switzerland) link was also housed at the end of the survey for participants to indicate their preferred availability for focus group participation. All participants provided electronic informed consent before scheduling focus group participation.

Once dates and assignments were confirmed, virtual focus group sessions were held via Zoom (San Jose, CA) on 3 separate dates dependent on the participants’ availability. Each focus group was limited to 4 participants. Participants were assigned to a waiting room upon entry, after which they were instructed to modify their name to a pseudonym of choice for the protection of privacy. The principal investigator (N.A.H.) and co-investigator (M.L.O.) served as session moderators who posed initial questions and prompted group discussion, modified questions to progress the conversation, and asked supplementary questions as needed. While participants answered each question individually, moderators encouraged interaction amongst the group to explore convergent and divergent perspectives. Each session was recorded with the verbal informed consent of participants via a transcription service embedded in the Zoom platform.

### 2.4. Data Analysis

The responses from the survey were collected using Qualtrics. All collected data were downloaded and transferred to SPSS (version 26; IBM Corp, Armonk, NY, USA) for analysis of descriptive statistics. Counts and percentages were used to summarize participant demographics. The mean interview duration was approximately 50 min. Interview transcriptions were proofed for accuracy against the recording by a third-party researcher with competency in qualitative data collection and management. Data analysis was approached using a 7-step process, as outlined by Charlick et al. [13]. The analysis process began by listening to audio in addition to reading and re-reading through the first transcript to gain a comprehensive understanding of the ATs’ experiences followed by a third reading which involved generating initial notes of free associations in the margins of the text. In vivo coding was used to place emphasis on the specific language used by the AT participants in support of the IPA design and the desire to report on the participants’ experiences. Portions of the transcripts and notes were analyzed for significant words and phrases used by the participants, and this language was developed into codes using spreadsheet software. Codes were then organized into initial emergent themes reflecting the meanings of the participants’ experiences. Researchers continued by seeking to identify connections between the themes. Honoring the individuality of each focus group, the analysis process was then repeated for the remaining two transcripts before patterns were identified across all three cases. The analysis was completed by the principal investigator (N.A.H.) and an external researcher, independently. When the transcript review was complete, the two analysts met as a team to discuss the results and reach a consensus on the identified themes. As a validity check, the principal investigator (N.A.H.) presented individual responses and common themes to participants as necessary to request any additions or deletions to improve the accuracy of the identified themes. Final themes were qualitatively summarized into results, and direct quotations were selected, as suggested by Pietkiewicz and Smith [14] and Pringle et al. [15], to give depth to the findings. Data credibility was established through member checking, multiple-analyst triangulation, and a peer review.

## 3. Results

Data saturation was reached at eleven participants after a redundancy in responses occurred in the third focus group session. Participants were 72.7% (*n* = 8) female and 27.3% (*n* = 3) male with an average age of 34.0 +/− 10.8 years. Regarding race/ethnicity, participants were 45.5% white- non-Hispanic/Latino (*n* = 5), 36.4.% Hispanic (*n* = 4), 9.1% Black (*n* = 1), and 9.1% (*n* = 1) American Indian or Alaska Native. Participants averaged 10.5 +/−10.8 years of athletic training certification with 7.6 +/− 7.9 years of overall practice and 7.3 +/− 7.9 years of practice at a Title 1 school. The demographics of individual participants can be found in Table 2.

### 3.1. Experiences Providing Primary Care Services in Title 1 Secondary Schools

Qualitative data from the focus groups revealed numerous SDoH affecting adolescent patients’ overall health, well-being, and qualities in addition to sometimes preventing or prolonging patients from receiving care for non-orthopedic health concerns (Table 3). These SDoH often affected participants’ practice as ATs. A distinctive and overarching theme emerged suggesting ATs in Title 1 schools internalized numerous roles related to helping their patients overcome SDoH, which served as a barrier to their access to quality healthcare. Additionally, key interrelated subthemes surfaced regarding the ATs’ roles including (1) role preparation, (2) role clarity, (3) facilitating patient-centered care, (4) limited integration of care, and (5) patient-centered strategies used to overcome access and quality barriers.

#### 3.1.1. Role Preparation

Participants described a range of educational experiences relevant to caring for non-orthopedic concerns. Of the 11 participants, half indicated their preparation for their role included some degree of formal general medicine coursework during their professional program or training. However, education was described as “broad”, “general”, and “non-specific”. The other participants drew knowledge from their prerequisite courses or sought out continuing education courses specific to primary care and non-orthopedic conditions that interested them. Several participants reported that the most valuable preparation for their role within their community was obtained informally through their interactions with physicians or other health providers. Likewise, and notably, two participants also discussed informal practice-based networks of AT colleagues. These networks shared issues (e.g., presentations of skin conditions) common to their community and patient population to exchange advice on how to proceed or navigate limited resources. Two others were employed through outreach (e.g., a physical therapy clinic) which offered regular professional development and annually reviewed skills such as auscultation. Of importance, two participants also reported no preparation or education relevant to primary care, although all noted the importance of this education to their daily practice and practice setting.

#### 3.1.2. Role Clarity

Participants self-identified with numerous roles associated with the primary care of their patients (Table 4). These roles included serving as a caregiver, care coordinator, advocate, and educator. In the caregiver role, participants often identified pre-existing health conditions or risk factors named by the patient’s parent or guardian and implemented preventive measures to protect the patient during sports participation. Likewise, ATs aimed to evaluate emergent health concerns or early warning signs of pathology, provide basic treatment, and initiate swift referrals. While ATs identified their role as a caregiver, all participants reported their primary role to be a navigator, or care coordinator, responsible for coordinating specialist care (e.g., dermatological, neurological, etc.), as they acknowledged more trained professionals or specialists were more likely to quickly address the root of the problem. When working as care coordinators, they collaborated with team physicians or school nurses to obtain needed care for non-orthopedic conditions. Participants also viewed themselves as patient advocates who ensured that patients and their families were able to navigate the healthcare system, obtain quality care, and understand the recommendations and information offered. Additionally important was the fact that ATs highlighted their roles as educators. When coordinating specialist care, participants also highlighted their desire to educate the parents and guardians of the patients and help them navigate the healthcare system. Moreover, as educators, they sought to provide their patients with advice on risk reduction and health promotion.

While participants were able to clearly self-identify these roles, they noted others (e.g., administrators, coaches, parents, etc.) were often confused about their role and responsibilities in the management of non-orthopedic conditions (Table 5). Participants identified that often individuals had a lack of clarity and understanding regarding the role and scope of practice of athletic trainers. In some cases, this role confusion resulted in delayed or missed opportunities for intervention with health concerns and underutilization of AT services. New students often held pre-convinced notions regarding athletic trainers’ willingness to help or intervene based on their past experiences with ATs at other schools or sporting events, avoiding encounters due to the assumption that the AT would not be willing or able to help. Furthermore, participants reported that despite the school administrators’ role as supervisors to athletic trainers, some lacked knowledge and understanding of the ATs’ scope of practice. This confusion resulted in the athletic trainers’ expectations being shifted toward forward-facing tasks (e.g., providing water and taping ankles) rather than patient care. Lastly, participants acknowledged parents and families as the ultimate decision-makers in an adolescent’s healthcare. However, some participants reported missed or underutilized opportunities for patient care resulting from parents’ ignorance or confusion about the athletic trainer’s role.

#### 3.1.3. Facilitating Patient-Centered Care

Reflecting on their own experiences, participants highlighted how their unique role within the schools and communities facilitated patient-centered care (Table 6). Direct access to the patients influenced the relationships built regarding trust, communication, and coordination of care. Positive, trusting relationships between patients and ATs can facilitate conversations and early interventions in general health concerns. Each participant emphasized the trust, respect, and rapport they were able to build with patients and some with patients’ families and communities. This trusting relationship meant patients were comfortable raising concerns to their ATs, often before family, physicians, or even school nurses in some cases. Furthermore, regular observation of and communication with patients through their proximity to athletics put ATs in a unique position to understand the athletes’ overall health, individual characteristics of expression (e.g., pain tolerances), and their unique social conditions. Thus, when unusual or persistent symptoms arose, ATs could identify what necessitated urgent referral and help the patient navigate the needed care. Moreover, their regular encounters with patients inside and outside of the athletic training facility allowed them to carefully watch over patients.

Additionally, participants felt their presence within the schools facilitated the coordination and integration of care with school counselors, nurses, and coaches to monitor student–athletes’ well-being, ensure basic needs were being met, and offer resources such as food or transportation. Collaboration also included the patients, as ATs emphasized their educational roles in health promotion, literacy, and navigation. Finally, the context of a student–athlete’s personal motivation to continue in a sport facilitates an exceptional adherence to the athletic trainer’s recommendations.

#### 3.1.4. Limited Integration of Care

Each participant reported referring patients to external providers. However, all participants also discussed obstacles to collaboration and communication among providers. Integration of care was restricted by numerous factors. One barrier was the high turnover of primary care physicians, which inhibited relationship building and ATs’ awareness of physicians’ accessibility to patients. A second barrier was the limited dedicated time in their workday to build relationships with physicians outside of school. A third barrier was the difficulty of navigating and staying up to date with healthcare resources (e.g., physicians who accept a variety of insurance plans, food distribution centers, and counseling) dispersed throughout a large school district or metro/rural area. A fourth barrier was the limited availability of specialty providers (e.g., dermatologists) despite the frequent occurrence of some non-orthopedic conditions.

#### 3.1.5. Patient-Centered Strategies to Overcome Access and Quality Barriers

Finally, some participants shared strategies to overcome patient- and community-specific barriers and support students’ access to primary care. Utilizing students’ sports physical forms was recommended as a mechanism to quickly identify active community-based primary care physicians who are accessible to their student–athletes (e.g., using a variety of insurance plans) and who are knowledgeable about their SDoH. Participants discussed proactively reaching out to these physicians to build a relationship for future referrals. Another strategy suggested schools or organizations employing ATs maintain a master list of area physicians in non-orthopedic specialties, like the initiatives used for referrals to orthopedic specialists. Lastly, participants advocated for the use of creative ways to work around the space constraints of their facilities, which prevent privacy, so that students feel comfortable to speak openly regarding SDoH or needed resources. Strategies include making themselves available for discussion during times when the athletic training facility is less busy or when walking to and from facilities or venues, and, additionally, providing students with discrete mechanisms (e.g., leaving a note on their desk) expressing a concern or desire to speak privately.

## 4. Discussion

Adolescents in low socioeconomic communities suffer from SDoH that serve as barriers to their healthcare access and quality. Thankfully, the presence of ATs in Title 1 secondary schools appears to be a protective factor for the student–athletes they serve. Athletic training services that are provided could lead to a reduction in health disparities for these adolescents that are often related to social determinants of health [16]. Therefore, ATs working in low socioeconomic communities should be prepared to ease the burden of barriers for adolescent student–athletes. The primary aim of this study was to describe the experiences of athletic trainers providing primary care services in low socioeconomic communities. Interesting and valuable findings emerged from the lucid reports of the participants. Although secondary school ATs largely focus on the management of sport-related injuries, this study demonstrated that they are, in fact, uniquely positioned to encounter general medical conditions as well. Athletic trainers in this study encountered various non-orthopedic conditions in the secondary school setting, most notably neurological and psychological conditions, which aligns with the recent foci of educational efforts related to the management of sport-related concussion and mental health in athletic healthcare. Patient encounters arising from other body systems were sparse; however, they were still reported by participants. We have reason to believe the ATs in our sample may have granted less recognition to the care they provide for non-orthopedic conditions and therefore underreported these patient encounters. For example, only two participants spoke about encounters arising from the respiratory system (i.e., asthma) even though participants were interviewed during the COVID-19 pandemic. Winkelmann and Games [17] reported that 28.2% of 611 ATs surveyed engaged in front-line screening or provided other AT services directly related to COVID-19. Screening activities can be rightfully classified as assessment, evaluation, or diagnosis, or, at minimum, risk reduction, and health promotion practices intended to avoid the spread of a respiratory illness. This suggests our AT participants may fail to classify these non-orthopedic services as general medicine or primary care. To the same point, when asked about the general medical conditions they encounter in their practice, no participants reported conditions arising from the genitourinary and gynecological systems. However, several ATs included within our sample described vivid lived experiences involving consultations with their student–athletes regarding reproductive health including menstrual cycle tracking and contraception as well as gender-affirming care. This further reinforces our suspicion that ATs may overlook these vital consultations as general medical services. While these findings may not be strong enough to extend to all Title 1 secondary school ATs or ATs as a whole, they warrant further investigation into how ATs define primary care and general medical conditions.

Even when non-orthopedic conditions are uncommon, ATs, particularly ones in low socioeconomic communities, play an integral part in inclusive healthcare not only in the management of these conditions but in the wellness practices and health literacy that student–athletes develop during their adolescent years. Being able to articulate the care provided for non-orthopedic conditions can not only improve ATs’ communication with external healthcare providers both in conversation as well as in appropriate medical documentation but can also give those external health care providers more confidence in viewing ATs as healthcare providers.

Education plays a vital role in preparing ATs for the roles and responsibilities of patient-centered care. The findings of this study suggested ATs sought education regarding general medical conditions through both formal and informal mechanisms. Many ATs received formal education regarding general medical conditions in their professional education, while others sought out the information in mandatory or voluntary continuing education sessions. We highlight the value of knowledge ATs obtained from their local physicians and within practice-based networks of colleagues. However, a lack of comprehensive formal education may result in a lack of confidence in managing these conditions. The 2020 CAATE Standards for Professional Programs [18] require that students receive didactic and/or clinical education regarding medical conditions originating from all major body systems. While the curricular content is being provided to the students, Bacon et al. [19] reported that as little as 3% of patient encounters recorded by AT students in clinical education were for non-orthopedic diagnoses. Limited clinical experience combined with knowledge decay occurring because of infrequent non-orthopedic encounters may lead ATs to exhibit deceased confidence when evaluating and treating these conditions. Acknowledging this deficiency, we believe there is a significant and obligatory need for continuing education in primary care that educates practicing ATs regarding general medical conditions, the best practices for the management of these conditions, in addition to the development and utilization of interprofessional relationships, which can help overcome access barriers to efficiency and effectively help lead adolescents of all backgrounds to quality healthcare services. Regardless of their education, experience level, and confidence, the ATs who were sampled felt they served in numerous roles that facilitated student–athletes’ overall health and well-being. Sociopsychological theories of personality related to self-identification suggest that individuals select and pursue goals in a way that supports or enriches the identities to which they are committed [20]. These roles included serving as a caregiver who triaged emergent conditions and initiated referrals, provided treatment within their scope, and offered social support, particularly for those with reduced healthcare access; a care coordinator who helped guide patients through the healthcare system; an educator who provided knowledge regarding risk reduction and health promotion; and an advocate who ensured needed resources and care were obtained.

While ATs were at least somewhat confident in the evaluation and treatment skills required to manage non-orthopedic conditions, the findings of this study suggest Title 1 ATs are most comfortable coordinating care for patients and advocating on behalf of their specific healthcare needs. Participants largely agreed that even in their caregiver role, their responsibilities lay not in the recognition and management of non-orthopedic conditions affecting the student–athletes’ overall health but in the coordination of care. In the care coordinator role, the ATs worked with patients and their families to understand their insurance coverage, find care providers who were accessible (e.g., translation services if needed, in a good location, and with convenient hours), and arrange their transportation. Athletic trainers reported using an interprofessional and collaborative approach, working with school counselors, nurses, and coaches to monitor student–athletes’ well-being and offer resources as necessary. Care coordination was viewed as the most important role, particularly when working with students and families with poor health literacy and/or inexperience with the healthcare system. Care models for these individuals should be centered around the patients and their individual circumstances. To provide patient-centered care, there must be continuity and integration of care between primary and specialty providers.

Social barriers exist which complicate care coordination and prohibit adolescents in low socioeconomic communities from receiving timely and effective primary care for non-orthopedic conditions. Athletic trainers at Title 1 schools were able to mitigate some access barriers for students–athletes attending their schools. Access to an AT alone reduces the negative effect of a lack of transportation on a patient’s health by reducing the need to seek healthcare outside of a school environment [9]. Furthermore, the unique position of ATs within schools and their athletic programs facilitated strong, trusting relationships to be established. Trust and respect are values inherent to quality, patient-centered health care. These relationships enabled easy reporting of orthopedic and non-orthopedic conditions by students as well as early recognition of general medical concerns by healthcare providers. Furthermore, ATs reported playing a crucial role in providing social support to their patients. Clement et al. [21] studied injured athletes’ perceptions of social support from peers, coaches, and ATs and found that social support from ATs had a significant effect on overall health and well-being. Therefore, building positive, supportive relationships with patients may help ATs promote health in their patients.

Although ATs largely focused on identifying orthopedic and non-orthopedic conditions that served as barriers to students’ athletic performances, their positions allowed them to distinguish more comprehensive conditions that spanned multiple body systems. Athletic trainers described both a desire and ability to mitigate some SDoH by managing certain general medical conditions in-house. In this sample, ATs were most commonly able to circumvent transportation and insurance barriers by performing post-operative rehabilitation in-house or using their trusting relationships to initiate initial conversations regarding food insecurity or mental health care. However, numerous barriers still endured when student–athletes needed specialty care outside of the ATs’ scope of practice. Despite changes resulting from the Affordable Care Act, nearly all ATs in this study cited insurance as the most challenging of these barriers. While all schools in this study required student–athletes to purchase an insurance policy, this policy could be utilized only for injuries and conditions resulting from their participation in sports, therefore failing to facilitate care for acute, underlying, or chronic conditions. Thus, ATs were forced to rely on other mechanisms such as free or community-based clinics to enable athletes to be seen by physicians. While tapping into their networks and their local resources was typically successful at helping patients to obtain needed services, the ATs’ roles in care coordination were acknowledged as difficult and time-consuming. The high turnover of primary care physicians within their communities inhibited relationship building and ATs’ awareness of physicians’ accessibility to student–athletes. Likewise, ATs felt they had limited time available to build relationships with physicians or other healthcare providers outside of school. Lastly, navigating and staying up to date with healthcare resources (e.g., physicians who accept particular insurance policies, procedures for obtaining translation or transportation resources, etc.) provided further frustration and time loss.

Approximately 80% of health outcomes are determined by factors other than medical care; therefore, it is important to be aware of how SDoH can positively or negatively contribute to the overall health of patients [16]. Furthermore, this information can be used to inform the best allocation of time, resources, and education in addressing non-orthopedic and orthopedic health matters. The presence of ATs in secondary schools may be a protective factor for the populations they serve and could lead to a reduction in health disparities that are often related to SDoH, such as income and access to care. Thus, efforts should be made to ensure ATs are provided to student–athletes in low socioeconomic communities, that these ATs are trained to practice at the top of their skillset, and that they are connected to a network of other healthcare providers for practice support and integration of care.

### Limitations and Future Research

Despite education on general medical conditions, we believe the participants from our sample did not fully understand the definition of primary care and as a result, may have underreported and undervalued important healthcare services in their qualitative reports. Thus, the frequency of non-orthopedic conditions encountered by ATs in this study may not accurately reflect the true rate of occurrence for these conditions. Additionally, we accept a missed opportunity to operationally define “primary care” and “general medical conditions” in the recruitment process and prior to the data collection.

While focus groups reached data saturation after 11 participants, using a convenience sample of participants in addition to word-of-mouth advertising presents concerns that the sample included was not fully representative of the population being studied. Thus, generalizations from this sample to all Title 1 secondary school ATs or all secondary school ATs, or ATs as a whole, should be applied with caution.

Future research should aim to determine how ATs define primary care and general medical conditions within their clinical practice. Furthermore, future studies should collect data regarding the frequency of non-orthopedic encounters in the secondary school setting and the relationship of that frequency to ATs’ confidence in managing general medical conditions as well as serving as primary care providers. Frequency data may serve as valuable in supporting the need for ATs in secondary schools. Additionally, ATs’ confidence in evaluating and treating these conditions should be assessed in an effort to identify continuing education opportunities for ATs regarding primary care conditions or specific body systems.

## 5. Conclusions

A greater understanding of primary care needs for adolescents in low socioeconomic communities requires the detection of their SDoH. Athletic trainers working at Title I secondary schools need to be aware of the social determinants affecting their student–athletes and the ability of those social determinants to affect overall student–athlete health and well-being. Because of their unique accessibility, Title 1 secondary school ATs are called upon to evaluate and treat student–athletes with general medical conditions. Often ATs need to refer these conditions to an appropriate healthcare provider when deemed outside of their scope of practice or confidence level. However, Title 1 ATs run into numerous, complex SDoH preventing efficient and effective referral to specialty health care providers. Thus, ATs ultimately felt their most important roles in primary care were as caregivers who mitigated avoidable barriers (e.g., insurance and transportation) by providing services in-house or within their referral network and care coordinators, which assisted student–athletes and their families with navigating the healthcare system (e.g., insurance, translation, etc.).

## Figures and Tables

**Table 1:** Focus group interview protocol.

What education have you had, if any, in non-orthopedic, primary care conditions?
2. Can you share a time in which you evaluated or provided treatment for a non-orthopedic condition or primary care concern? What was the nature of the condition? Was this a new or pre-existing condition?
3. Can you think of any instances where you needed, or would have preferred, to refer a patient for a primary care concern? What barriers existed to referral? How did you overcome these barriers? What were you most worried about during this encounter?
4. Can you think of any instances where a patient failed to seek your help or care for a non-orthopedic condition or primary care concern? What was the result of this encounter? Did they share why they did not see you first?
5. Can you describe the established relationships that you have with other healthcare professionals (e.g., physicians, nurses, etc.) for the management of non-orthopedic conditions or primary care concerns? How were these relationships established? What barriers exist to working as an interprofessional team? What barriers exist to establishing interprofessional relationships?

**Table 2:** Participant demographics.

Pseudonym	Age	Gender	Race or Ethnicity	Years of Certification	Years in Secondary School	Years in Title 1 School
Abby	28	Female	Hispanic	2	1.5	1.5
Duker	60	Male	White	35	27	27
NewMom	30	Female	White	8	8	8
Iris	32	Female	Hispanic	7	7	7
Ashley	26	Female	White	4	4	4
Rick	46	Male	Hispanic	23	17	17
RM	41	Female	White	19.75	6	5
Mackenzie	29	Female	White	3.5	2.25	2.25
Marco	26	Male	Hispanic	1	1	1
Reese	30	Female	Black	8	8	6
Mickey	26	Female	AI/AN	4	2	2

AI/AN American Indian or Alaska Native.

**Table 3:** Social determinants of health.

Determinant	Supporting Quotation
Economic stability	“She said I don’t have insurance […] they [the patient and their family] were kicked off of extra assistance because brother got a job, a part-time job, that is not enough to support the family.”—Mackenzie “Parents just don’t wanna take time off of work. Maybe they can’t… They feel like their jobs might be jeopardized, sometimes there’s even immigration where you’re talking to somebody who might not be legal, legally documented, and so they don’t wanna go be a part of the bureaucratic paperwork giving over their names, revealing maybe they don’t have the proper documentation.”—Rick “I don’t wanna say they [patients] feel prideful or they feel shameful about it, but they struggle to be able to admit like I can’t access food.”—NewMom
Education access and quality	“Language is a huge barrier towards getting appropriate care and referrals […] Some of my patients won’t get translation services that they need because their language is too specialized.”—RM
Healthcare access and quality	“The biggest barrier is insurance, especially since it changes every year and some students have limited coverage or are on Medicaid.”—Ashley “We [AT staff] wanted her to get a surgery with a certain physician because it was a sport specialized Ortho, but she couldn’t [due to insurance issues] so she ended up getting a surgery with another doctor and she ended up having two surgeries because the rehab and everything was not was not the best for her, but because of her insurance, she had to go there and she literally struggled with it more than what should have been for her injury.”—Ashley
Neighborhood and built environment	“We also deal with asthma, my schools by an airport, and so depending upon flight patterns and which field I am at can have more [asthma] flare-ups, so really being conscientious of the social determinants of health from environment.”—RM “A lot of gun violence and community violence where I’m from.”—Mickey “Gun violence is prevalent in our community as well […] we’ve lost several students to gun violence, street gangs and suicide, we’ve had kids go missing.”—RM

**Table 4:** Identified roles in primary care of the patients.

Role	Supporting Quotation
Caregiver	“I think that our job with non-orthopedic injuries is to identify those are things that are at risk during physicals […] and also to monitor those [patients] who are at high risk or have a pre-existing condition.”—Abby “I focus on recognizing signs and symptoms [of non-orthopedic condition] and then providing maybe counseling”—RM “Gosh, I always snag at least four kids a year for hypertension monitoring and education, particularly in my black and African-American students as well as my Samoan population”—RM
Care coordinator	“I feel like we’re the starting point for all of that, they come to us and then we do not push them off but instead say why don’t you go talk to this counselor about what you just told me. […] I just feel like we are the starting point for these kids to get those [needed resources].”—Mackenzie “It’s what is best for the athlete, obviously we’re going to provide patient-centered care. […] I’m not an expert on a lot of things, so I’m gonna look for that expert that can help me and assist my athlete to get to where we need to be.”—Iris “I work with our coaching staff to be like, this kid’s parents can’t take them to the doctor, if I set up the appointment, are you available to take them there? I do quite a bit of insurance work with my parents and guardians.”—Mickey
Advocate	“We’re the frontline. Obviously, we can Google someone but I don’t feel like any of us would be feel comfortable just sending our kids off to someone that we just looked up on the Internet. If we know and trust these people [other health care providers] were gonna send them there.”—Mackenzie “I attend as many patient visits as I can, just because I like to be an advocate for my patient […] for the parent or guardians who may not be able to navigate that as seamlessly as someone with my background can.”—Mickey “I’m making sure that those people [patients and parents] actually get the care that I sent them there for, which is a big challenge, especially in my Spanish and Vietnamese speaking populations.”—RM
Educator	“What I did was educate him [diabetic athlete] on what he should be eating, how frequently to talk to his doctor to make sure that the medication he was taking was controlling his blood sugar […] just helping him out with the educational part, which was, I think the most difficult for him.”—Abby “A lot of reproductive work [education] around periods and just contraceptive use, that kind of thing.”—Mickey “So I have a lot of misuse with inhalers, a lot of time with my kids a lot [is devoted to education], so that’s something that I kinda have to combat every year with the kids coming in from middle school and younger populations.”—Ashley

**Table 5:** Role confusion.

Concept	Supporting Quotation
Standard of care	“It is a revolving issue with students transferring from other school […] there’s some athletes that come to you and when you tell them ‘No, it’s an issue,’ or they’re surprised you do something that another [athletic] trainer didn’t do.”—Reese
Perception	“I got a new principal two years ago, and he observed pre-season practice one day, and the thing he was most concerned about was that I didn’t have a towel over my shoulder, a water bottle in my hand, and I wasn’t squirting water in their mouth […] I’ve been lucky that I’ve had athletic directors that understand my job, but obviously we have some people that still don’t understand fully what we do.”—Iris “Just the history for us as athletic trainers working in secondary schools, it’s kind of hard, they don’t know all that we do besides taping, evaluations or if you know you get hurt on and off the field.”—Reese “I think it’s lack of education on that aspect and what they see on a daily basis that we do, that’s what they think… Those are our duties, but they don’t realize that we have the ability to work with all these general medical conditions as well.”—Abby
Scope of practice	“I believe there are times when a parent truly doesn’t understand what the athletic trainer does despite our best efforts [e.g., parent orientation meetings], and they feel that their child is best served by going to primary, going to urgent care, going to ED [emergency department].”—Mickey
	“I think it’s mostly because they don’t know or they’re not aware that we can actually treat those kind of conditions. Um obviously, we focus more in orthopedics […] they may be not thinking that we’re capable of helping them in that situation.”—Iris

**Table 6:** Strategies for facilitating patient-centered care.

Strategy	Supporting Quotation
Establishing rapport	“I don’t know if I had any formal training in being able to recognize it [lacerated spleen] other than just knowing this individual and knowing and seeing the incident happen and just recognizing that this was not right.”—Marco
Building trust	“I always advocate for myself, let the parents know that they can trust me in helping the healing process with their child.”—Reese
Treating with respect	“I think this is the best way to teach them about healthcare—through us—respecting their autonomy and having it be self-driven care. I can help facilitate that and meet their goals.”—RM
Being available	“I feel like going out to practice and just having very nonchalant conversations with people gives students sometimes an opportunity to be like ‘hey, when it’s not super busy, can I talk to you about this one thing’ and address an existing question or concern.”—NewMom
Providing comfort	“I’m gonna offer this [resource] before you even have to say anything so that you know you feel comfortable talking with me about it.”—NewMom
Staying vigilant	“I think our main goal is really to be vigilant to not invalidate someone’s symptoms or feelings when they do come to us with something that is non-orthopedic. The main thing is really just being vigilant, knowing who our athletes are, knowing where they come from, and understanding them.”—Marco

## Data Availability

Not applicable.
